# Supplementary material for: A Predictive Model of Adaptive Resistance to BRAF/MEK Inhibitors in Melanoma
Source: Int J Mol Sci. 2023 May 7;24(9):8407. doi: 10.3390/ijms24098407 (PMC10178962; doi:10.3390/ijms24098407)
Supplement: Supplementary file 1 [file ijms-24-08407-s001.zip › ijms-2356620-supplementary.pdf]

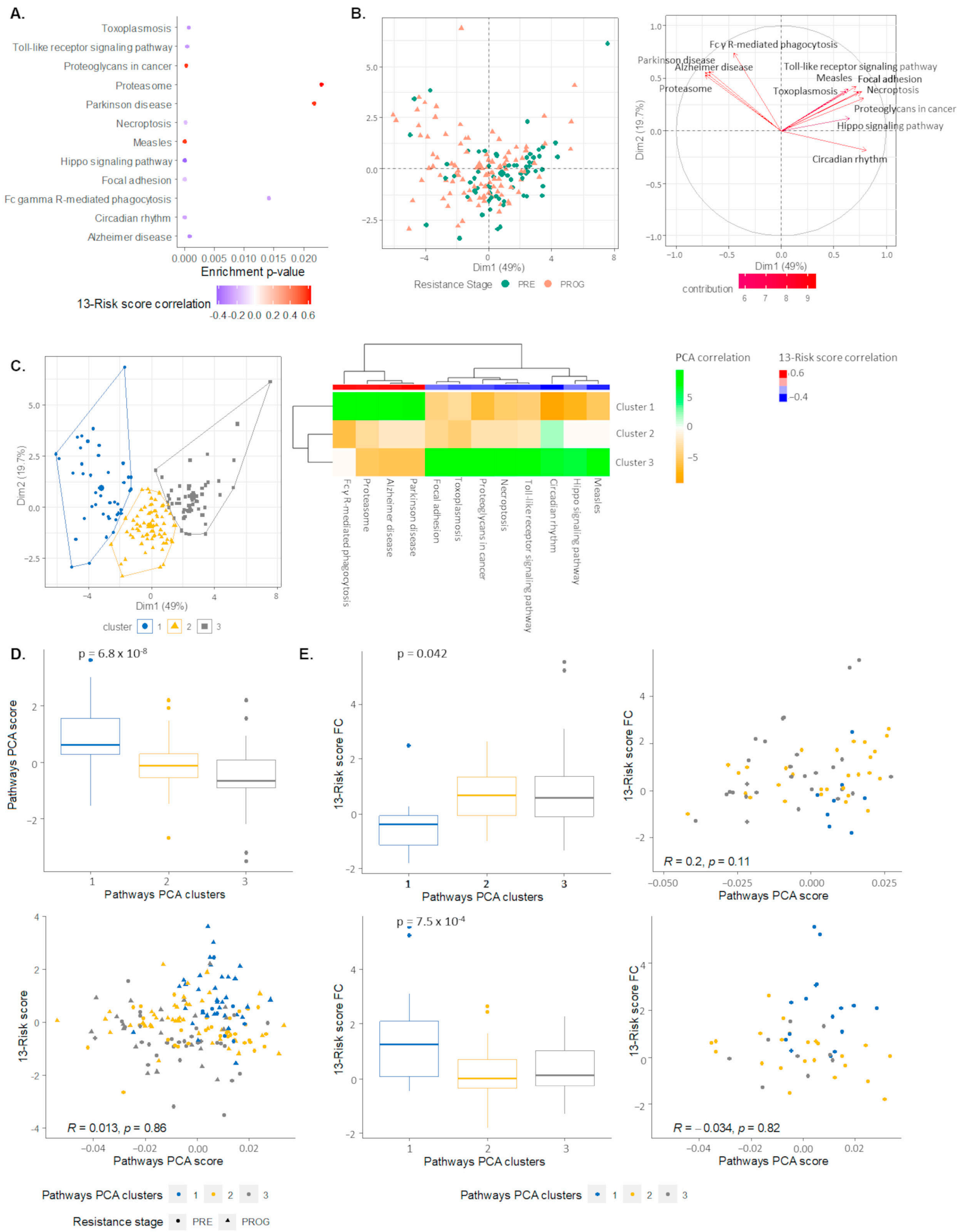

Figure S1: A subset of pathway signaling are 13-risk score-dependent to modulate therapeutic sensitivity of human melanoma FiveDatabases samples. (**A**). Dotplots representing the enrichment p-value of the 12 pathways that present a similar significant correlation with the 13-risk score in the GSE99898 and FiveDatabases samples. (**B**). Principal component analysis (PCA) of the 13 pathways' scores (**right**) explaining the variance observed in the GSE99898 samples (**left**), differentiated by their resistance stage. (**C**). Hierarchical clustering of the previous PCA identifying three clusters of samples (**left**) and pathway's score according to the clusters samples (**right**). (**D**). A 13-risk score expression according to the pathways PCA clusters (**top**) and score (bottom) in the samples. (E) 13-risk score fold change (FC) according to the pathways PCA clusters (**left**) and score (**right**) in the PRE

(**top**) and PROG (**bottom**) samples. Kruskal-Wallis p-value (significant < 0.05) for boxplots, spearman correlation coefficient with corresponding p-value for dotplots.

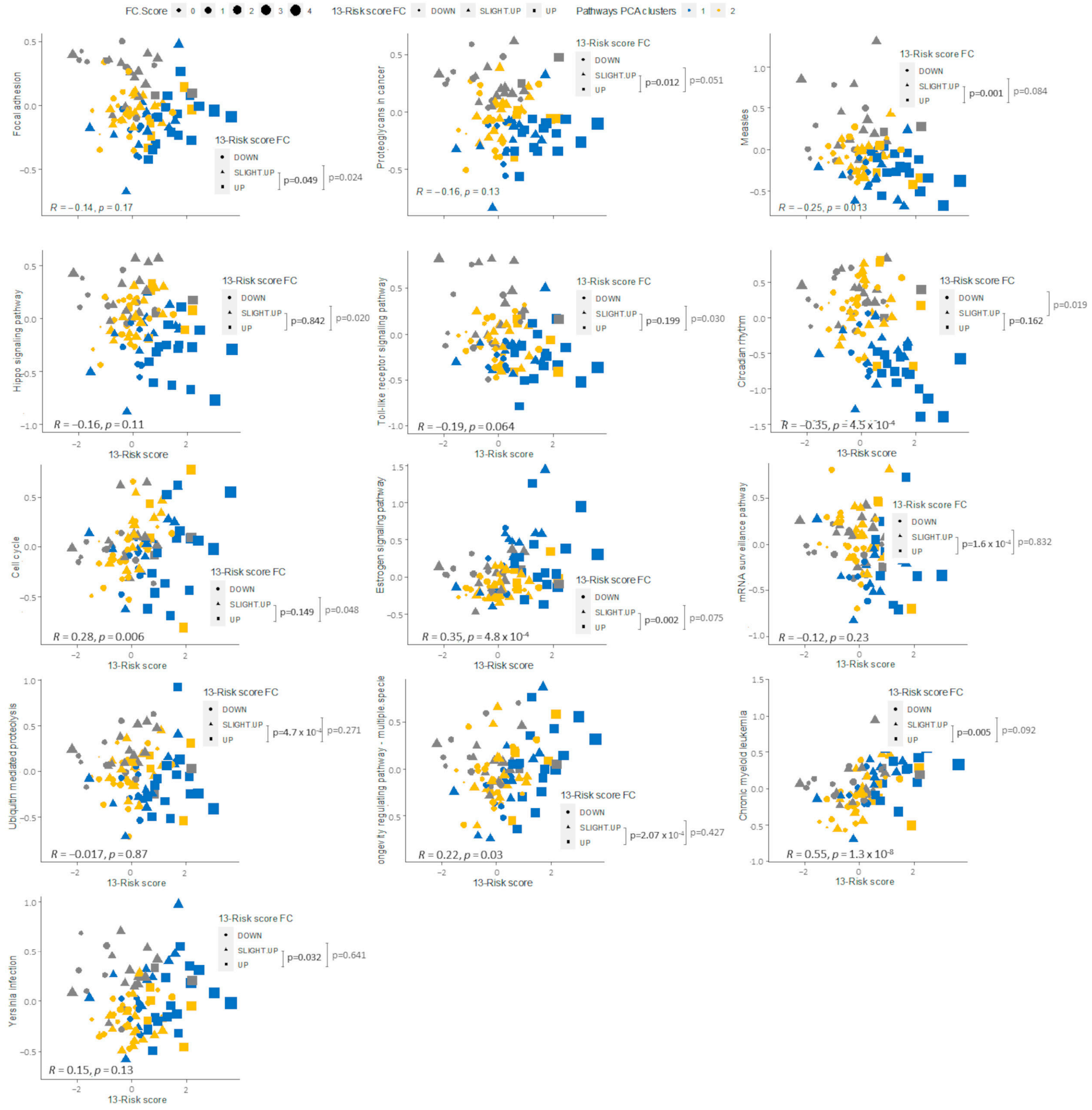

Figure S2: Pathways score distribution according to the 13-Risk score and the 13-risk score fold change (FC), stratified as downregulated, slightly upregulated, and upregulated, in the PROG FiveDatabases samples. Spearman's correlation with corresponding *p*-values and Kruskal-Wallis test for the 13-Risk score FC comparison (*p*-value significant < 0.05).
